# Pd(II)‐Catalyzed Strategies for the β‐Arylation of α‐Amino Acids: An Overview

**DOI:** 10.1002/chem.202502272

**Published:** 2025-10-07

**Authors:** Davide Illuminati, Claudio Trapella, Vinicio Zanirato, Virginia Cristofori, Anna Fantinati

**Affiliations:** ^1^ Department of Environmental and Prevention Sciences University of Ferrara Via Luigi Borsari Ferrara 46, 44121 Italy; ^2^ Department of Life Sciences University of Modena and Reggio Emilia Via G. Campi 213/d Modena 41125 Italy; ^3^ Department of Chemical, Pharmaceutical and Agricultural Sciences University of Ferrara Via Fossato di Mortara 17 Ferrara 44121 Italy

**Keywords:** amino acids, arylation, late‐stage functionalization, ligand design, palladium catalysis

## Abstract

Metal‐catalyzed C─H activation has been linked to amino acids for decades. Peptides and α‐amino acids (α‐AAs) have been used as substrates, directing groups (DGs) and ligands, significantly expanding and broadening the definition of metallo‐ and organo‐catalysis. This mini‐review focuses on strategies for β‐arylation of α‐AAs involving Pd(II)‐mediated activation of the β‐C(sp^3^)‐H sigma bond. After a brief introduction in Section 1, strategies exploiting auxiliary bidentate DGs to address the Pd(II)‐mediated C─H activation are discussed in Section 2. The related Ligand‐controlled processes are treated in Section 3 and Section 4 highlight the most recent atom‐economic developments in peptide late‐stage functionalization (LSF) proceeding *via* Pd(II)‐mediated β‐C(sp^3^)‐H activation of a precise α‐AA residue along the peptide sequence.

## Graphical abstract

The graphical abstract summarizes the review “Pd(II)‐Catalyzed Strategies for the β‐Arylation of α‐Amino Acids: An Overview”, highlighting representative Pd(II)‐catalyzed methods for the β‐arylation of α‐amino acids. Key mechanistic features, substrate diversity, and synthetic relevance of these transformations are showcased.

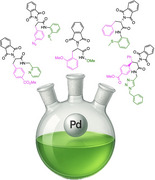

## 1 Introduction

From the first discovery and clinical application of insulin for the treatment of type I diabetes, a remarkable rise of peptide drugs has been registered, especially since the late 1980s. With the continuous expansion of our understanding of the functions of proteins and enzymes, it is reasonable to expect important biochemical results. Consequently, it is not surprising that peptide synthesis remains an expanding field of research.^[^ ^1^, ^2^, ^3^, ^4^ ^]^ Peptides and peptidomimetics are not only relevant in medicinal chemistry and therapeutics; they also find different applications in material sciences, proteomics, diagnostics, and drug discovery.^[^ ^5^, ^6^, ^7^, ^8^, ^9^, ^10^ ^]^

This trend has been driven by the maturation of peptide synthesis technologies, increased investments and research efforts in therapeutic peptide market.

Among the wide world of peptides, nonproteinogenic amino acids, peptidomimetics, peptoids, and peptides containing nonnatural amino acids gained significant attention over the past decades. This is largely due to their improved biological activity and pharmacokinetic properties compared to their parent peptides.^[^ ^11^, ^12^, ^13^, ^14^, ^15^, ^16^ ^]^

Nonnatural amino acids, for instance, have shown remarkable potential in improving biological activity, receptor affinity and selectivity. In the field of opioid peptides, even the replacement of a single amino acid can have an enormous effect. A notable example is the incorporation of the 2,6‐dimethyl‐tyrosine (Dmt), at different positions within opioid peptides, which has led to both predictable and unexpected outcomes.^[^ ^17^, ^18^, ^19^, ^20^, ^21^, ^22^ ^]^

Notably, nonnatural α‐AAs can also be exploited as useful building blocks in total synthesis,^[^ ^23^, ^24^, ^25^ ^]^ as well as chiral ligands and catalysts.^[^ ^26^, ^27^, ^28^ ^]^

Over the years, many different methods to synthesize nonnatural α‐AAs have been reported, starting from asymmetric Strecker reactions,^[^ ^29^, ^30^, ^31^ ^]^ enantioselective hydrogenation of dehydroamino acid precursors,^[^ ^32^, ^33^ ^]^ asymmetric alkylation of Glycine derivatives through chiral auxiliaries,^[^ ^34^, ^35^, ^36^, ^37^, ^38^ ^]^ to the use of transition metals (TMs) to insert or modify side chain residues by C─C, C─N, and C─O bond forming reactions.^[^ ^39^, ^40^, ^41^, ^42^ ^]^

Since the introduction of aryl substituents on the side chain of aliphatic α ‐AAs is one of the most valuable synthetic strategies to gain nonproteinogenic derivatives, many different approaches have been reported in the literature.^[^ ^43^, ^44^, ^45^, ^46^ ^]^ Cross‐coupling reactions involving inert C(sp^3^)‐H activation as the initial step effectively allowed different side chain modified α ‐AAs to be prepared with C α stereo‐retention. In general, TM‐catalyzed processes leading to the formation of new C─C sigma bonds *via* inert C─H activation use simple, inexpensive, and minimally prefunctionalized building blocks, managing to transform them into more complex structures through short and therefore more environmentally sustainable synthesis processes.

In this context, one of the first example of palladium‐catalyzed cross‐coupling reaction on an inert C─H bond was reported by Murahashi^[^ ^47^ ^]^ in 1979, subsequently exploited in 1984 by Tremont^[^ ^48^ ^]^ to produce *ortho*‐alkylated acetanilides. This area of research was further developed by Daugulis^[^ ^49^ ^]^ and Chatani^[^ ^50^ ^]^ who highlighted the advantages of using DGs to facilitate the key C(sp^3^)‐H activation step.

This mini‐review highlights the evolution of synthetic methodologies for β‐arylation of α‐AAs, focusing on particular on Pd(II)‐mediated activation of inert β‐C(sp^3^)‐H sigma bonds.

The most relevant studies have been organized in Sections 2, 3 and 4 as follows. Section 2 deals with strategies exploiting auxiliary bidentate DGs to achieve the Pd(II)‐mediated C─H activation‐arylation in position β. Section 3 describes related methods defined as Ligand‐controlled or Ligand‐enabled β‐C(sp^3^)‐H activation‐arylation processes. Eventually, Section 4 discusses recent atom‐economic developments in peptide late‐stage functionalization (LSF) proceeding *via* Pd(II)‐mediated β‐C(sp^3^)‐H activation‐arylation of a specific α ‐AA residues within the peptide sequence.

**Scheme 1:**
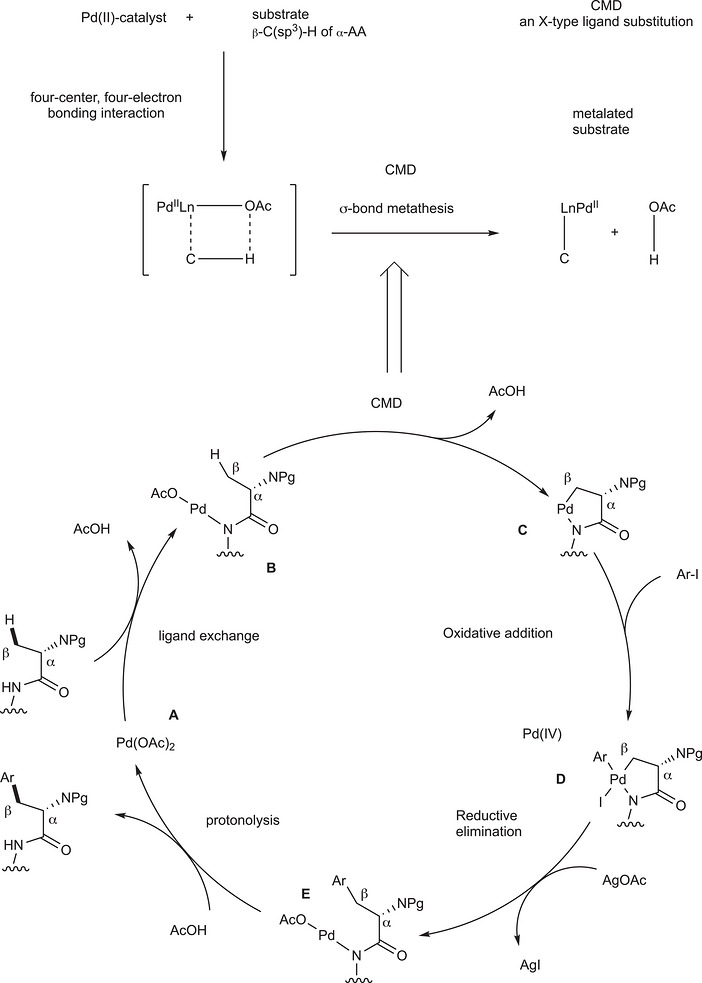
(top) Pd(II) catalyzed C─H activation *via* concerted metalation‐deprotonation (CMD), (bottom) General Pd(II)/Pd(IV) CHF cycle.

Regarding β‐C(sp^3^)‐H activation, a four‐center, four‐electron bonding interaction between the Pd(II)‐catalyst and the target C─H sigma‐bond of the substrate has been proposed. This interaction leads to a sigma‐bond metathesis process (top of Scheme 1) also referred to as concerted metalation‐deprotonation (CMD), which corresponds to an X‐type ligand substitution facilitated by the carboxylate ligand (OAc). The resulting carbon‐centered palladium‐complex, (**C** in Scheme 1) enables the formation of a new sigma C─C bond through a cross‐coupling reaction.

Overall, the C─H functionalization (CHF) of single AAs (see Section 2 and 3) or of a specific AA residue in a peptide sequence (see Section 4) follows the basic steps shown in Scheme 1. An initial ligand exchange step, usually assisted by DG and/or ligands converts the catalyst (**A**) into catalyst (**B**) in which the metal center is located near the β‐C(sp^3^)‐H bond to be activated. At this point, the critical site‐selective β‐C(sp^3^)‐H bond activation step takes place according to the sigma bond metathesis process mentioned above (Scheme 1), to give (**C**).

Oxidative addition of (**C**) onto the Ar‐I sigma bond, a step usually facilitated by the presence of silver or copper salts as additives to remove iodide anion, produces (**D**). The unstable high‐valent Pd species (**D**) undergoes reductive elimination to (**E**), which forms the desired β‐arylated α ‐AA and the Pd(II) catalyst (**A**) via protonolysis. (Scheme 1).

## 2 Pd‐Cat. β‐Srylations of A‐AAs Using Amide‐Linked Bidentate Directing Groups^[^ ^49^, ^50^, ^51^, ^52^, ^53^, ^54^, ^55^, ^56^ ^]^

Generally, an effective DG should:

- remain stable under reaction conditions adopted for the required functionalization,
- avoid direct participation in the reaction,
- coordinate weakly and reversibly to the metal catalyst,
- be easily removable at the final stage of synthesis.

Most of this section is devoted to the illustration of methods that exploit β‐C(sp^3^)‐H arylation through the use of *N*,*N*‐bidentate DGs. Particularly, subsection 2.1 collects DGs belonging to the amino *N*‐heterocycles (ANH) class, subsection 2.2 those belonging to the aminomethyl *N*‐heterocycles (AMNH) and subsection 2.3 is dedicated to aliphatic dimethylaminoethylamine (DAEA) directing group. Instead, in subsection 2.4 have been included different methods for the β‐C(sp^3^)‐H arylation through both *N*,*O*‐ and *N*,*S*‐bidentate directional groups, namely glycine dimethylamide (GMDA), and 2‐thiomethylaniline (TMA), respectively.

All the bidentate auxiliary groups herein presented cooperate with Pd(II) to affect the ancillary C(sp^3^)‐H activation by which Pd(II)‐intermediate complexes (**C**) are formed (Scheme 1).

For the benefit of the reader, in the following Schemes the structure of the key 5,5‐bicyclic Pd(II)‐intermediate complex (**C**) has been reported within square brackets.

### 2.1 Methods Using DGs of the Amino *N*‐heterocycles (ANH) Class

**Scheme 2:**
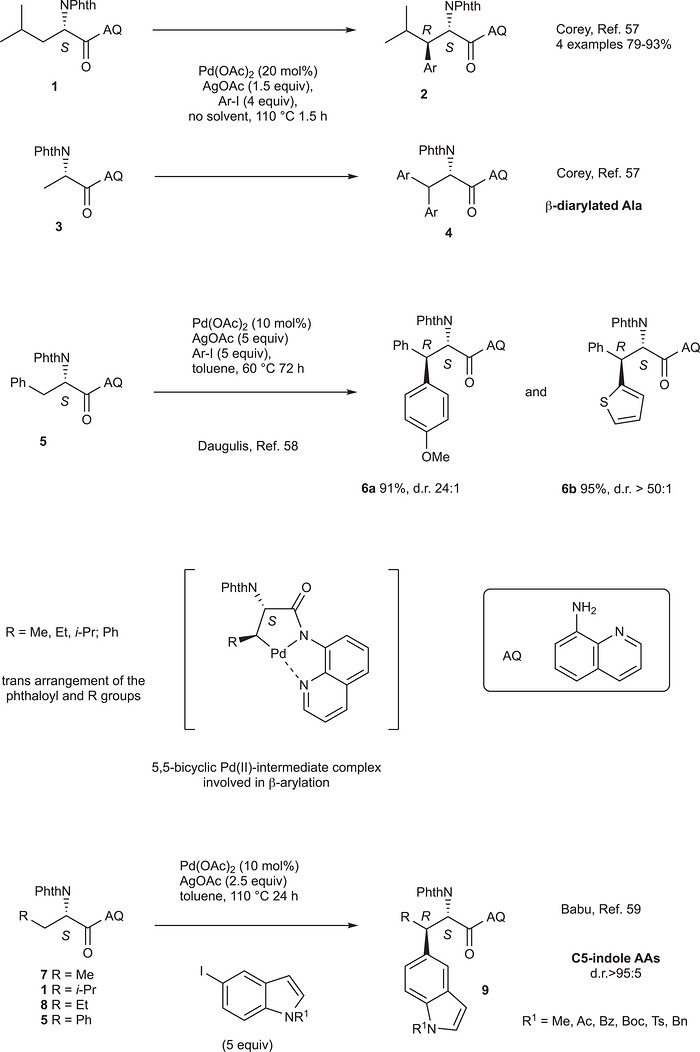
Examples of Pd(II)‐catalyzed β‐C(sp^3^)‐H arylations of Phth‐AA‐AQ.

Corey et al. observed for the first time the secondary β‐C(sp^3^) arylation of *N*‐phthaloyl Leucine prefunctionalized by amidation with 8‐aminoquinoline (AQ). Under optimized conditions, [Pd(OAc)_2_ (20 mol%), AgOAc (1.5 equiv), Ar‐I (4 equiv), heating at 110 °C for 1.5 hours], Phth‐Leu‐AQ (**1**) gave the corresponding β‐arylated AA‐derivative (**2**) with good *anti*‐stereoselectivity (Scheme 2). Interestingly, Phth‐Ala‐AQ (**3**) submitted to the same reaction conditions afforded the bis‐arylated derivative (**4**) in good yield.^[^ ^57^ ^]^

Subsequently, by lowering the reaction temperature to 70 °C, Daugulis and coworkers succeeded in the monoarylation of Phth‐Phe‐AQ (**5**) obtaining the *anti*diastereomers (**6a**) and (**6b**) in good yield and high diastereoselectivity. (Scheme 2).^[^ ^58^ ^]^

Both Corey and Daugulis postulated that the observed diastereoselectivity arised from the formation of a sterically more favored *trans*‐palladacycle intermediate (Scheme 2). Thus, the diastereoselectivity of arylation is established in the palladium‐catalyzed C─H activation (CMD) step, while the subsequent reductive elimination step (Scheme 1) is assumed to proceed with retention of configuration. This well‐established stereochemical result of secondary β‐C(sp^3^)‐H arylation reactions recently enabled Babu et al.^[^ ^59^ ^]^ to transform prefunctionalized norvaline (**7**), leucine (**1**), norleucine (**8**) and phenylalanine (**5**) into unnatural C‐5 indole AA derivatives of general formula (**9**) with good diastereoselectivity (*anti*, d.r. > 95:5) and enantiopurity (e.r. up to 99:1).

**Scheme 3:**
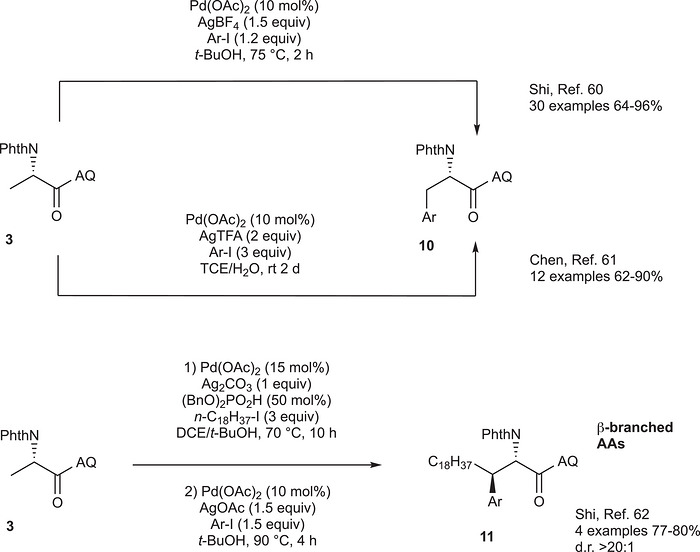
Examples of Pd(II)‐catalyzed β‐C(sp^3^)‐H monoarylations and sequential alkylation‐arylation of Phth‐Ala‐AQ.

The problem of monoarylation versus double arylation of Phth‐Ala‐AQ (3) was addressed independently by the Shi^[^ ^60^ ^]^ and Chen^[^ ^61^ ^]^ groups (Scheme 3). The former prepared derivatives of general formula (10) by heating the reaction mixture in tert‐butanol, using AgBF4 instead of AgOAc, the latter prepared (10) by carrying out the reaction at room temperature in a biphasic system of 1,1,2,2‐tetrachloroethane (TCE) and H2O, using AgTFA as an additive. Furthermore, Shi succeeded in finding the reaction conditions to produce β‐branched α‐amino acids (11) by performing sequential alkylation‐arylation of Phth‐Ala‐AQ (3) (Scheme 3).^[^ ^62^ ^]^

**Scheme 4:**
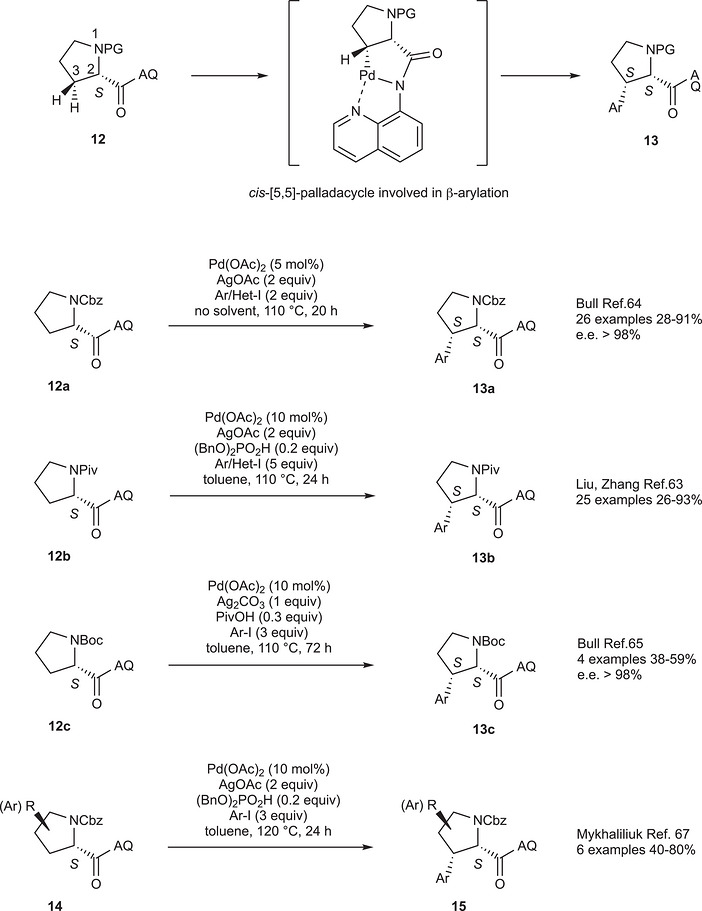
Pd‐catalyzed β‐C(sp^3^)‐H arylation on *N*‐protected Pro‐AQ.

The AQ‐guided Pd(II)‐catalyzed β‐C(sp^3^)‐H arylation has been expanded to cyclic amino acids in the last decade.^[^ ^63^, ^64^, ^65^, ^66^, ^67^ ^]^ For example, proline‐derivatives (**12**) equipped with the AQ directing group have been arylated with a wide range of iodoaryl derivatives, including some heteroaryl ones, to give (**13**) (Scheme 4). It should be noted that heteroaryl iodides often turn out to be catalyst poisoners due to the highly coordinating properties of the heteroatoms they contain.

*Cis*‐2,3‐disubstituted pyrrolidines were formed as single stereoisomers through putative *cis*‐[5,5]‐palladacycles originating from AQ's preferential orientation toward the C(3)‐H at the *cis*‐position to the carboxamide group (Scheme 4). Substiuents at C(4) or C(5) of the proline ring are usually tolerated if they occupy a *trans*‐position to the carboxamide group (see conversion (**14**) to (**15**)). Different protections for the secondary amine have been tested: the *N*‐Boc derivatives showed reduced propensity to the Pd‐catalyzed *cis*‐C(3) arylation compared to the corresponding *N*‐Cbz derivatives. Extensive research was required to define the best reaction conditions in terms of catalyst loading, operating temperature, solvent type, and the presence of acidic additives such as dibenzyl phosphate (DBP) or pivalic acid. (Scheme 4). However, cleavage of the AQ directional group proved to be highly resistant to a wide variety of reagents. A major advantage to the method^[^ ^64^ ^]^ resulted from the use of 5‐methoxy‐8‐aminoquinoline (MQ) as an equally effective auxiliary group, readily removable under oxidative conditions using cerium ammonium nitrate (CAN) or cerium ammonium sulfate (CAS)^[^ ^68^ ^]^.

(For a more convenient method of removing the AQ auxiliary group see [ref. 69]).

**Scheme 5:**
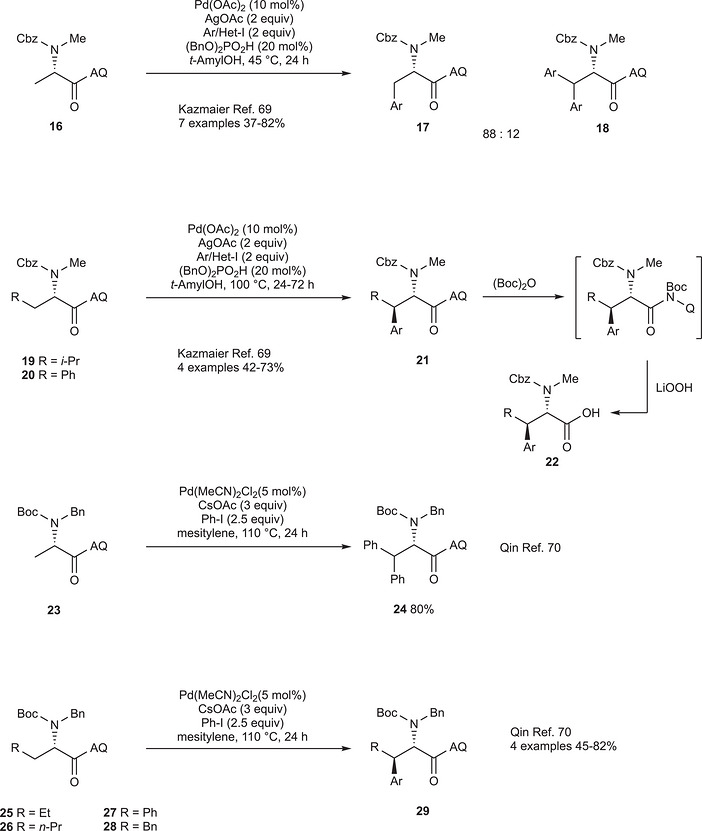
Pd‐catalyzed β‐C(sp^3^)‐H arylation on Cbz‐(*N*‐Me)AA‐AQ and Boc‐(*N*‐Bn)AA‐AQ.

Peptides incorporating unnatural *N*‐methylalanine derivatives hold great promise in drug discovery due to their increased resistance to proteolysis and increased permeability. A paper published by Kazmaier and Kinsinger in 2018 fits into this context.^[^ ^69^ ^]^ They discovered an efficient method for the Pd‐catalyzed stereoselective β‐functionalization of prefunctionalized *N*‐methyl AAs. In particular, they demonstrated that Cbz‐(*N*‐Me)Ala‐AQ (**16**) was suitable for modification by Pd(II)‐catalyzed primary β‐C(sp^3^)‐H activation‐arylation using a wide range of aryl and heteroaryl iodides (Scheme 5). The reported optimized conditions were: heating the substrate and ArI (2 equiv) in *t*‐AmylOH at 45 °C for 24 hours in the presence of Pd(OAc)_2_ (10 mol%), DBP (20 mol%), AgOAc (2 equiv).

Under these conditions, the mono‐/bis‐arylate ratio (**17**)/(**18**) was 88:12, and the reactions proceeded without epimerization. It is worth noting that alkyl and alkenyl iodides were also usable, albeit with limited success. The protocol was further applied to the dipeptide Cbz‐Leu‐(*N*‐Me)Ala‐AQ, affording exclusively the β‐C(sp^3^)‐H arylation‐hetarylation of the (*N*‐Me)Ala residue in comparable yield to the results obtained with the single amino acid (see sub‐section 4.1, scheme 23).

Finally, it has been shown that CHF of secondary β‐C(sp^3^)‐H is also possible, with both Cbz‐(*N*‐Me)Leu‐AQ (**19**) and Cbz‐(*N*‐Me)Phe‐AQ (**20**) being readily transformable into the derivatives of general formula (**21**) (Scheme 5).

It should be noted that the removal of AQ was easily achieved by converting the arylated products (**21**) into the corresponding *N*‐Boc‐protected analogues which, through subsequent saponification with LiOOH, afforded the compounds of general formula (**22**). However, the minor formation of doubly arylated by‐products represented a limitation as it necessitated challenging purification procedures.

The β,β‐diarylation of Boc‐(*N*‐Bn)Ala‐AQ (**23**) to give (**24**) and the β‐monoarylations of Boc‐(*N*‐Bn)AA‐AQ (**25**–**28**) to give derivatives of general formula (**29**) were instead reported by Qin and colleagues (Scheme 5).^[^ ^70^ ^]^ The method appears original in the panorama of Pd(II)‐catalyzed β‐C(sp^3^)‐H functionalization of AA derivatives as it does not require the presence of silver salts as iodide scavengers. In fact, the reaction conditions involved heating the prefunctionalized AAs and Ar‐I (2.5 equiv) in mesitylene at 110 °C for 24 hours in the presence of Pd(MeCN)_2_Cl_2_ (5 mol%), CsOAc (3 equiv). The use of silver salts has been avoided, simplifying the reaction treatment and reducing waste production.

**Scheme 6:**
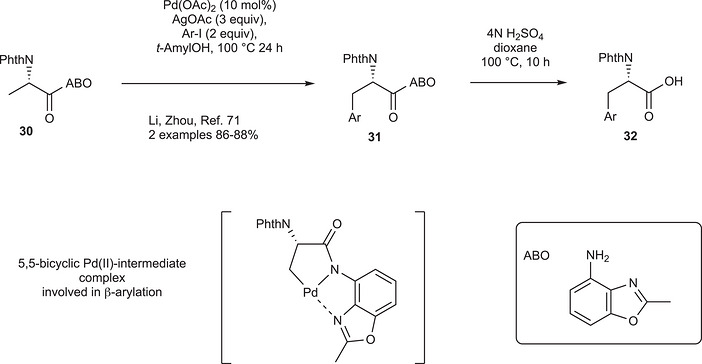
Pd‐catalyzed β‐C(sp^3^)‐H monoarylation on Phth‐Ala‐ABO.

2‐Methyl‐7‐amino benzoxazole (ABO) has been used as an alternative DG from the amino *N*‐heterocycles (ANH) group. Thus, phthaloylalanine equipped with 2‐methyl‐7‐amino benzoxazole (ABO) Phth‐Ala‐ABO (**30**) gave good results in the conversion to the monoarylated derivatives of general formula (**31**) by heating in *t*‐amyl‐OH at 100 °C for 24 hours using the mixture of Pd(OAc)_2_ (10 mol%), AgOAc (3 equiv), Ar‐I (2 equiv).^[^ ^71^ ^]^ Subsequent hydrolysis under acidic conditions provided the corresponding Phth‐AA‐OH (**32**) in an excellent yield (Scheme 6).

4‐Aminobenzotriazole (ABTA), an ANH directing group recently introduced by Song,^[^ ^72^ ^]^ has the advantage of being easily removable. The optimized conditions for the arylation of Phth‐Ala‐ABTA (**33**) to give derivatives of the general formula (**34**) were: heating the substrate and Ar‐I (3 equiv) in hexafluoroisopropanol (HFIP) at 80 °C, under Ar atmosphere for 24 hours, in the presence of Pd(OAc)_2_ (10 mol%), Ag_2_CO_3_ (3 equiv).

**Scheme 7:**
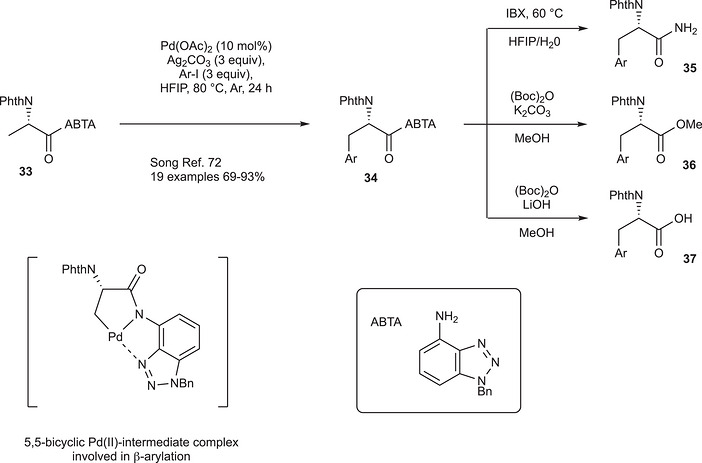
Pd‐catalyzed β‐C(sp^3^)‐H monoarylation on Phth‐Ala‐ABTA.

The ABTA‐assisted primary β‐C(sp^3^)‐H mono‐arylation maintained high efficiency regardless of the type of substituted aryl iodides employed (Scheme 7). To remove the auxiliary ABTA directing group from (**34**) there are three options available. First, an oxidation with iodoxybenzoic acid (IBX) to give the carboxamides (**35**), second, the conversion to the esters (**36**) by base‐promoted methanolysis of the corresponding Boc‐imide derivatives, and third, the saponification of the latter to give the carboxylic acid derivatives (**37**).

### 2.2 Methods Using DGs of the Aminomethyl *N*‐heterocycles (AMNH) Class

**Scheme 8:**
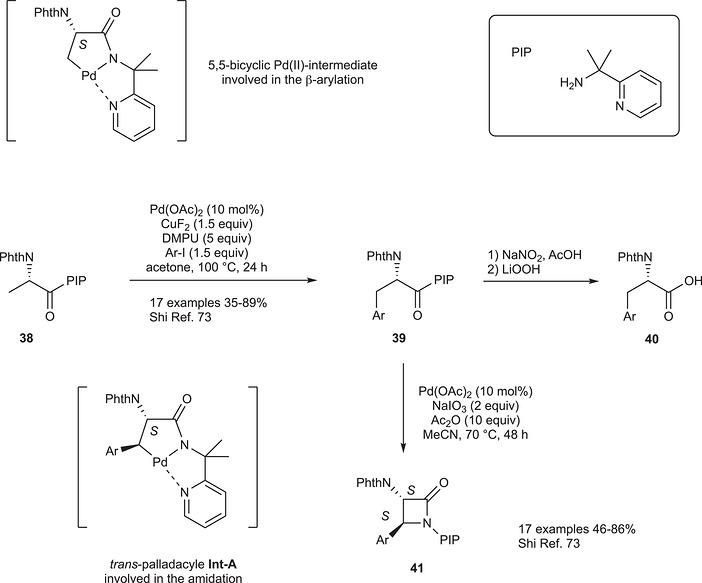
Pd‐catalyzed β‐C(sp^3^)‐H monoarylation on Phth‐Ala‐PIP.

The use of 2‐(pyridin‐2‐yl)‐isopropyl amine (PIP) as well as 2‐(2‐pyridylethyl)‐amine (PE) and 2‐pyridylmethyl‐amine (PM) as useful *N,N*‐bidentate DGs has also been reported. Intriguingly, these DGs strictly required phthaloyl *N*‐protection of the amino acid, in fact, the arylations did not occur in the case of *N*‐carbamate protection. Thus, Phth‐Ala‐PIP (**38**) has been shown to be an optimal substrate to carry out primary β‐C(sp^3^)‐H monoarylations, also with sterically hindered aryl iodides.^[^ ^73^ ^]^ The optimal reaction conditions for the preparation of compounds (**39**) include the presence of *N*,*N’*‐dimethylpropyleneurea (DMPU) as the coordinating solvent to promote monoarylation, while the catalyst system is Pd(OAc)_2_ (10 mol%) and CuF_2_ (1.5 equiv) (Scheme 8). Removal of the PIP auxiliary group can be accomplished by heating under acidic conditions or by a mild *N*‐nitrosylation/hydrolysis sequence which gives (**40**) in high yield.

Interestingly, the monoarylated compounds (**39**) were cyclized into chiral α ‐AA‐β‐lactams (**41**) under oxidative conditions (NaIO_3_/Ac_2_O) in the presence of Pd(OAc)_2_. It has been proposed that intramolecular C(sp^3^)‐H amidation proceeds via the five‐member *trans*‐palladacycle **Int‐A** (Scheme 8). From the latter intermediate, the release of the β‐lactams (**41**) likely involves oxidation to Pd(IV) to promote stereoretentive C─N reductive elimination.

**Scheme 9:**
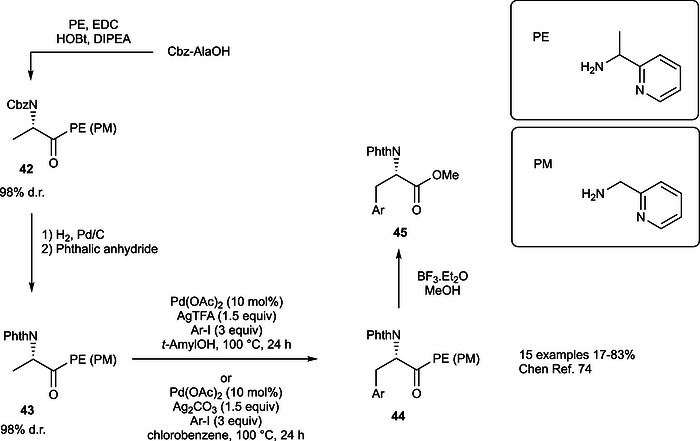
Pd‐catalyzed β‐C(sp^3^)‐H monoarylation on Phth‐Ala‐PE(PM).

Few years later, Chen and coworkers succeeded in the palladium‐catalyzed monoarylation of Phth‐Ala‐PE(PM) (**43**) by performing the C─C coupling reactions in *t*‐amyl alcohol/AgTFA or in chlorobenzene/Ag_2_CO_3_ (Scheme 9).^[^ ^74^ ^]^

The authors reported that in the preliminary stage of equipping Phth‐Ala‐OH with PE using EDC or HATU‐mediated amidation systems, significant racemization at C α of Ala was recorded. This complication was overcome by performing the amidation on Cbz‐AlaOH and subsequently by exchanging the Cbz group of (**42**) with Phth to obtain diastereomerically pure (**43**). Finally, PE cleavage from (**44**) was performed via BF_3_^.^Et_2_O promoted methanolysis to produce (**45**).

**Scheme 10:**
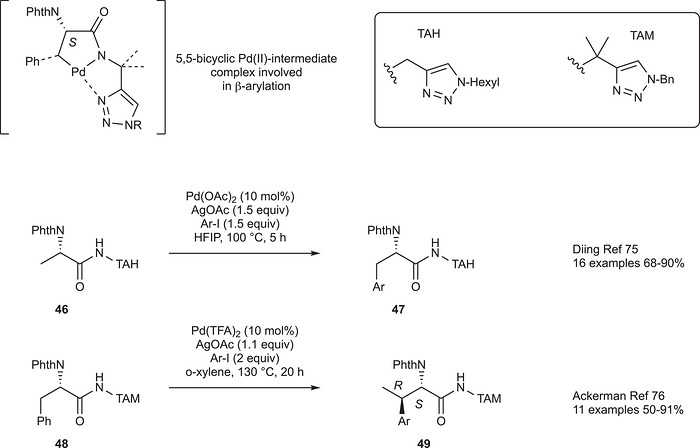
Pd‐catalyzed β‐C(sp^3^)‐H monoarylation on Phth‐Ala‐TAH and Phth‐Phe‐TAM.

Click 1,2,3‐aminotriazoles as triazolylmethylhexyl (TAH) and triazolyldimethylmethyl (TAM), easily accessible through 1,3‐dipolar Huisgen‐like cycloadditions, have been introduced as convenient DGs for high yielding stereospecific and diastereoselective DG‐guided Pd(II)‐catalyzed β‐C(sp^3^)‐H arylation of amino acid derivatives (Scheme 10). For example, Ding research group used Phth‐Ala‐TAH amide (**46**) to insert a variety of aryl substituent bearing either electron‐withdrawing or electron‐donating groups including *ortho*‐substituted derivatives.^[^ ^75^ ^]^ The best reaction conditions for the preparation of the monoarylated derivatives (**47**) were: heating the substrate and Ar‐I (1.5 equiv) in hexafluoroisopropanol (HFIP) at 100 °C for 5 hours in the presence of Pd(OAc)_2_ (10 mol%), AgOAc (1.5 equiv).

Subsequently, Ackerman described both primary and secondary Pd(II)‐catalyzed β‐C(sp^3^)‐H arylations using TAM as a directing group. Thus, the amide Phth‐Phe‐TAM (**48**) underwent diastereoselective secondary β‐C(sp^3^)‐H arylations with broad Ar‐I spectrum and without racemization at the stereogenic centers (Scheme 10).^[^ ^76^ ^]^ The best reaction conditions for the preparation of derivatives (**49**) were: heating the substrate and Ar‐I (2 equiv) at 130 °C for 20 hours in *o*‐xylene in the presence of Pd(TFA)_2_ (10 mol%), AgOAc (1.1 equiv).

The removal of the TAH and TAM DGs occurred via BF_3_‐promoted methanolysis.

Notably, the TAMs *N,N*‐bidentate DG has also been used in site‐ and regio‐selective modification of peptide residues, opening to LSF (see Section 4, scheme 25).

### 2.3 2‐Dimethylaminoethylamine (DAEA) as an *N*,*N*‐bidentate DG

**Scheme 11:**
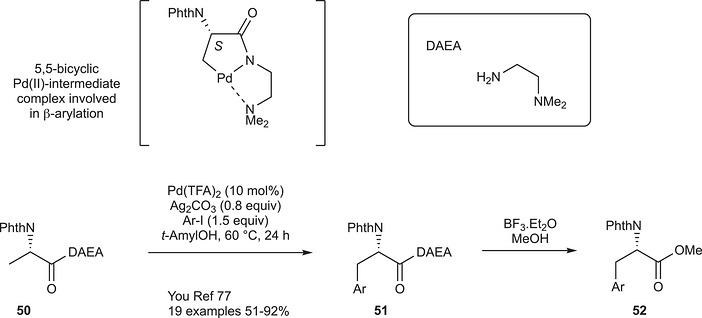
Pd‐catalyzed β‐C(sp^3^)‐H monoarylation on Phth‐Ala‐DAEA.

In 2018, You et al.^[^ ^77^ ^]^ reported the use of 2‐Dimethylaminoethylamine (DAEA) as a simple, cheap, readily available, easily removable and atom‐economical directing group for C─H functionalization (Scheme 11). For example, Phth‐Ala‐DAEA compound (**50**) allowed facile access to the corresponding β‐arylated derivatives (**51**) by using the Pd(TFA)_2_/Ag_2_CO_3_ system in *t*‐AmOH at 60 °C under air atmosphere. Interestingly, the aliphatic diamine auxiliary was not effective toward the arylation of secondary β‐C(sp^3^)‐H bond. Finally, DAEA was readily removed from (**51**) *via* BF_3_‐promoted methanolysis, giving the methyl esters (**52**).

### 2.4 Methods Using *N*,*O*‐ and *N*,*S*‐bidentate DGs

**Scheme 12:**
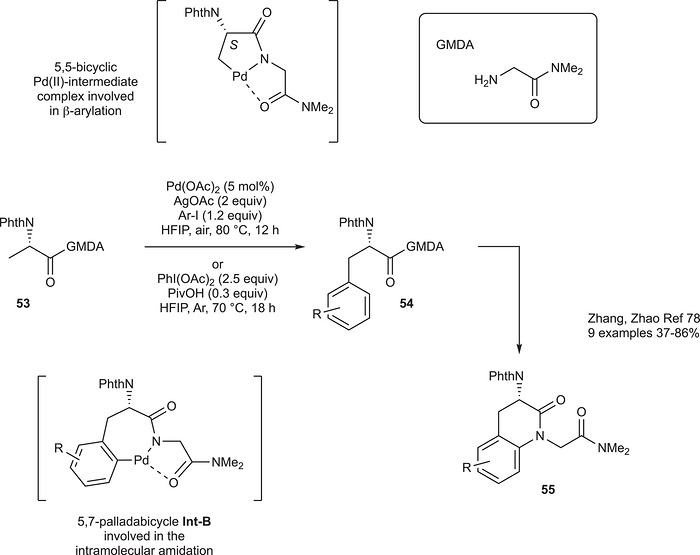
Pd‐catalyzed cascade C─H functionalization on Phth‐Ala‐GMDA.

Glycine dimethylamide (GMDA) has been used as an *N,O*‐bidentate directing group assisting the Pd(II)‐catalyst to prime mono‐arylation of phthaloylalanine (Scheme 12). The conversion of compound (**53**) to (**55**) has been achieved in high yield by heating the rection mixture in HFIP in air atmosphere or in the presence of the oxidant PhI(OAc)_2_. Clearly, the initially formed β‐arylated compounds (**54**) undergo subsequent Pd‐catalyzed intramolecular amidation. The two‐step C─H monoarylation/amidation sequence resembles that previously described in Scheme 8,^[^ ^73^ ^]^ however, in this case, a 5,7‐palladabicycle **Int‐B** is involved in the production of the substituted 2‐quinolinone derivative (**55**).^[^ ^78^ ^]^ Undoubtedly, the tandem β‐C(sp^3^)‐H arylation and δ‐C(sp^2^)‐H intramolecular amidation shown in Scheme 12 is a convenient one‐pot, two‐step route to afford bioactive compounds key intermediates. As a point of weakness, the removal of the GMDA auxiliary directing group occurred in strong acidic conditions (concentrated HCl at 100 °C).

**Scheme 13:**
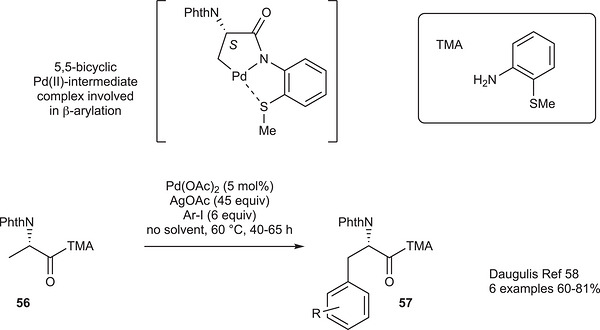
Pd‐catalyzed CHF on Phth‐Ala‐TMA.

Phthaloylalanine equipped with the 2‐thiomethylaniline (TMA) auxiliary, compound (**56**) in Scheme 13, gave good results of mono‐arylation under classical reaction conditions.^[^ ^58^ ^]^ The decreased reactivity of TMA in comparison with AQ directing group allowed a selective monoarylation. No side products formation has been reported. However, partial racemization at C α was sometimes registered while removing the DG auxiliary group from (**57**) through BF_3_‐promoted methanolysis.

The scope of the method was recently expanded to include 3‐ and 4‐iodoacetanilide as arylating species to prepare photo‐responsive azobenzene‐based unnatural amino acids.^[^ ^79^ ^]^

**Scheme 14:**
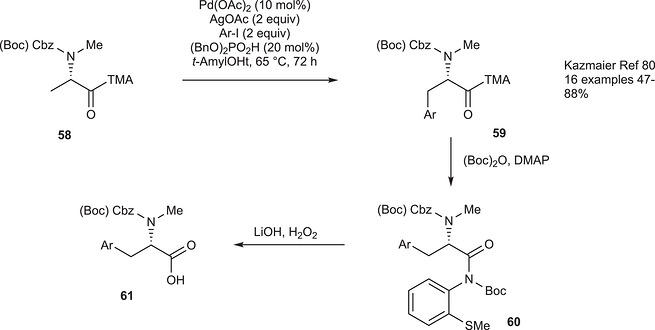
Pd‐catalyzed CHF on Cbz‐(*N*‐Me)Ala‐TMA.

As previously stated,^[^ ^57^, ^69^ ^]^ the AQ‐directed arylations of primary β‐C(sp^3^)‐H bonds do not stop after the first modification step but result in double‐functionalized products. This problem was brillantly solved by Kazmaier^[^ ^80^ ^]^ using TMA as the directing group (Scheme 14). The optimal conditions involved heating the substrate and Ar/(Het)‐I (2 equiv) in *t*‐amylOH at 65 °C for 72 hours in the presence of Pd(OAc)_2_ (10 mol%), AgOAc (2 equiv), (BnO)_2_PO_2_H (20 mol%). Indeed, Boc/Cbz‐(*N*‐Me)Ala‐TMA (**58**) was found to be suitable for the monoarylation process using a variety of aryl and heteroaryl iodides including *N*‐Boc 5‐iodoindole. Free hydroxyl and aldehyde groups were also tolerated, and the auxiliary TMA could be removed from (**59**) using conditions previously demonstrated to be effective for AQ removal.^[^ ^69^ ^]^ Thus, *N*‐Boc protection of the β‐monoarylated products afforded compounds (**60**) which when hydrolyzed with LiOOH afforded the corresponding free carboxylic acids (**61**) in excellent yields. Notably, this protocol has also been applied to introduce highly functionalized side chains into the C‐terminal alanines of dipeptides (see sub‐section 4.1, scheme 26).

## 3 Pd(II)‐Catalyzed Ligand‐Controlled β‐C(sp^3^)‐H Activation‐Arylation of α ‐AAs

This section reviews studies that led to the development of suitable Pd(II) ligands to give selectivity to the two‐step one‐pot activation‐arylation process of primary or secondary β‐C(sp^3^)‐H bonds in weakly coordinating AA‐substrate.

Subsection 3.1. is dedicated to strategies using AAs equipped with monodentate DGs while in subsection 3.2. have been included protocols that do not require the installation of exogenous DGs on the AA substrates.

### 3.1 Ligand‐Controlled β‐Arylation of AAs Equipped with Monodentate DGs

**Scheme 15:**
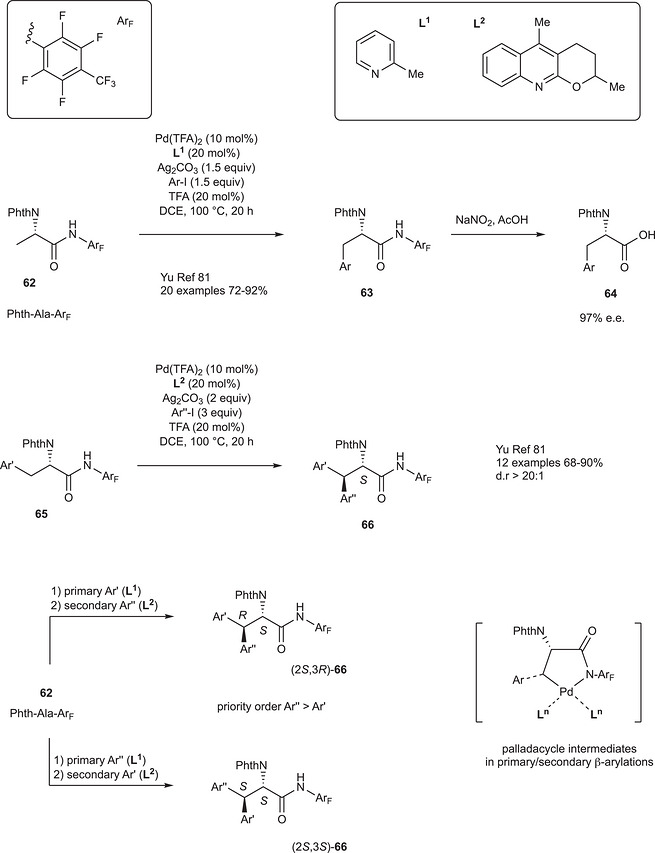
Pd(II)‐catalyzed **L^1^/L^2^**‐enabled β‐C(sp^3^)‐H mono‐ and diarylation on Phth‐Ala‐Ar_F_.

Yu's group^[^ ^81^ ^]^ reported the use of 2,3,5,6‐tetrafluoro‐4‐(trifluoromethyl)aniline (Ar_F_) as a convenient substrate‐bound auxiliary DG in cooperation with the 2‐picoline ligand (L^1^) to perform the exclusive primary β‐C(sp^3^)‐H (*ortho*‐permissive) monoarylation of Ala (Scheme 15). Thus, Phth‐Ala‐Ar_F_ (62) was transformed into derivatives (63) in good yields with no racemization at C‐ α. Optimized conditions for the monodentate DG/Ligand cooperative arylation were: Pd(TFA)_2_ (10 mol%), (L^1^) (20 mol%), ArI (1.5 equiv), Ag_2_CO_3_ in DCE at 100 °C. The reaction mixture was required to contain trifluoroacetic acid (TFA) to prevent base‐promoted substrate decomposition.

Removal of the DG by nitrosation (NaNO_2_, AcOH/Ac_2_O) readily afforded the corresponding carboxylic acids (64) with optimal enantiopurity.

In the same study, it was also reported that 2,5‐dimethyl‐3,4‐dihydro‐2H‐pyrano‐[2,3‐*b*]‐quinoline, ligand (L^2^) displayed favorable steric and electronic properties for catalyst activation toward the arylation of secondary β‐carbons of *N*‐protected α ‐AAs (65) to give diarylated derivatives (66). Successive application of these ligands enabled the sequential diarylation of a methyl group in an alanine derivative with two different aryl iodides, providing a wide range of β‐Ar’‐β‐Ar’′‐ α ‐AAs with excellent diastereoselectivity (Scheme 15). Specifically, (L^1^) was first employed to achieve monoarylation, followed by the use (L^2^) to perform the secondary β‐C(sp^3^)‐H arylation with a threefold excess of a distinct aryl iodide. This strategy allowed access to both configurations of the β‐chiral center in (61) simply by changing the installation order of the two aryl groups.

**Scheme 16:**
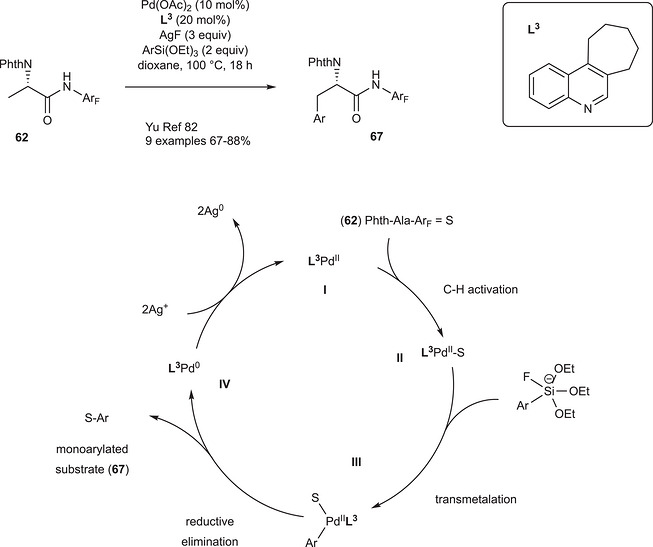
Pd(II)‐catalyzed **L^3^**‐enabled β‐C(sp^3^)‐H monoarylation on Phth‐Ala‐Ar_F_ with arylsilanes.

The quinoline‐based ligand (**L^3^**) (20 mol%) was crucial for the Pd(II)‐catalyzed cross‐coupling of Phth‐Ala‐Ar_F_ (**62**) with arylsilanes to give compounds (**67**) (Scheme 16). Successful cross‐coupling reactions with a broad range of electron‐rich and electron‐poor triethoxyarylsilanes (2 equiv) were achieved by heating the substrate in dioxane, employing Pd(OAc)_2_ (10 mol%) as the catalyst in the presence of AgF (3 equiv).^[^ ^82^ ^]^ The latter, according to the authors, plays a dual role in this Hiyama‐type cross‐coupling reaction: (1) silver salts are among the most efficient and commonly used oxidants to reoxidizing Pd(0) to Pd(II) in Pd(II)/Pd(0) catalytic cycles, and (2) fluoride sources are known to activate organosilicon coupling partners, thereby facilitating transmetalation of aryl groups to Pd(II). Unlike the previously described Pd(II)/Pd(IV) catalytic cycle, in this case a Pd(II)/Pd(0) catalytic cycle is presumably involved (Scheme 16). In the proposed mechanism, starting from intermediate (**I**), the (**L^3^**)/Ar_F_ directed β‐C(sp^3^)‐H activation of (**62**) affords intermediate (**II**), which undergoes transmetalation with triethoxyarylsilane to generate the Pd(II)‐intermediate (**III**). Reductive elimination from (**III**) releases the cross‐coupling product (**67**), while forming Pd(0) species (**IV**). Finally, reoxidation by Ag^+^ rigenerates (**I**), the active Pd(II)‐catalyst.

**Scheme 17:**
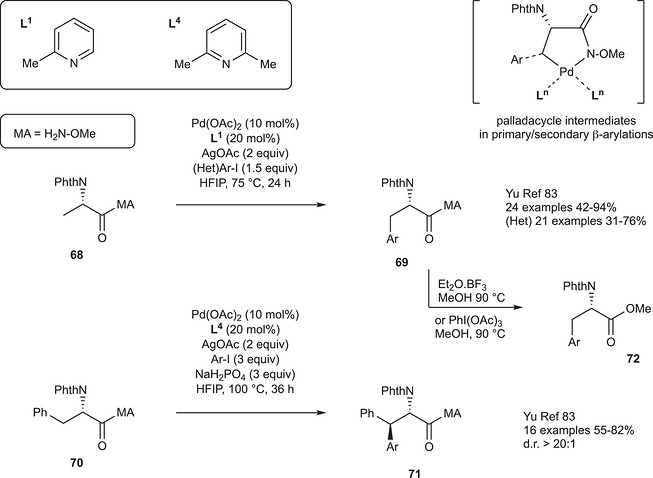
Pd(II)‐catalyzed **L^1^/L**4‐enabled β‐C(sp^3^)‐H arylations of Phth‐Ala/Phe‐MA.

Yu's group identified methoxylamine (MA) as an effective auxiliary group for β‐C(sp^3^)‐H arylation of carboxylic acids lacking α ‐Hs. The same research group later developed new conditions for the β‐monoarylation of Phth‐Ala‐MA and Phth‐Phe‐MA.^[^ ^83^ ^]^ In fact, it was discovered that 2‐Picoline ligand (**L^1^**) promoted the selective monoarylation of primary C(sp^3^)‐H bonds, whereas 2,6‐lutidine ligand (**L^4^**) enabled the arylation of secondary C(sp^3^)‐H bonds (Scheme 17). The optimized conditions for primary CHF of Phth‐Ala‐MA (**68**) to afford compounds (**69**) involved heating the susbtrate and ArI (1.5 equiv) in HFIP at 75 °C in the presence of Pd(OAc)_2_ (10 mol%), (**L^1^**) (20 mol%), AgOAc (2 equiv). Substituting (**L^1^**) with (**L^4^**) enabled the transformation of Phth‐Phe‐MA (**70**) into (**71**) via secondary CHF, in this case adding a triple amount of Ar‐I and the additive NaH_2_PO_4_ (3 equiv). The arylations were carried out in HFIP which, as an acid solvent, prevented the decomposition of the MA amides. Moreover, sequential arylation of alanine derivatives with two different aryl iodides (Ar^'^‐I and Ar^‘’^‐I) using these ligands enabled the introduction of two distinct aryl groups to produce a variety of β‐Ar^'^‐β‐Ar^''^‐α‐amino acids with excellent diastereoselectivity. In terms of results, the new method appears to mimic those described in Schemes 15, however, the MA DG introduced the practical advantage of being an easily installable and removable auxiliary group compared to Ar_F_. A wide range of variously substituted aryl and heteroaryl iodides were compatible, including those bearing common DGs such as acetamide, phosphonate, and hydroxyl moieties. Auxiliary removal was efficiently achieved either by BF_3_‐mediated methanolysis or by treatment with iodosobenzene diacetate [PhI(OAc)_2_] in methanol at 80 °C, both of which furnished the corresponding methyl esters (**72**) without racemization at the α‐chiral center.

**Scheme 18:**
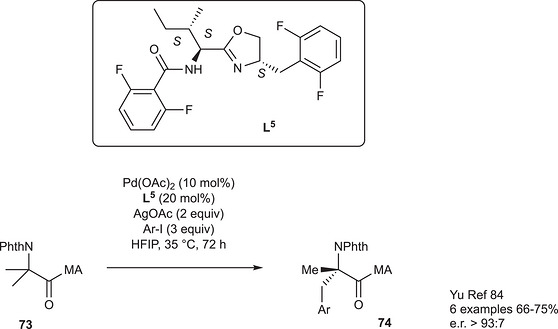
Pd(II)‐catalyzed **L^5^**‐enabled enantioselective β‐C(sp^3^)‐H arylation of Phth‐Aib‐MA.

The same group,^[^ ^84^ ^]^ a few years later, succeeded in the desymmetrization of α ‐aminoisobutyric acid (Aib) by converting Phth‐Aib‐MA (**73**) into nonracemic chiral derivatives (**74**) through ligand‐enabled β‐arylation (Scheme 18). In the pretransition state of the reaction, the chiral aminomethyloxazoline ligand (**L^5^**) exposes only one of the enantiotopic methyl groups of Aib to the dual activation‐arylation step mediated by Pd(II).

The reaction with a variety of aryl iodides afforded otherwise difficult‐to‐access enantioenriched α, α ‐disubstituted α ‐amino acid derivatives. The optimized desymmetrization protocol consisted of heating the substrate and ArI (3 equiv) in HFIP at 35 °C for 72 hours in the presence of (**L^5^**) (20 mol%), Pd(OAc)_2_ (10 mol%), AgOAc (2 equiv).

Both the N‐methoxy amide auxiliary and the Phth protecting group were removed and converted into Fmoc‐protected AAs, which represent valuable building blocks for peptide drugs.

### 3.2 Ligand‐Controlled DGs‐free β‐Arylation of AAs

The main disadvantage of the approaches previously described in sub‐sections 2.1–2.4 and 3.1 is that they inevitably require separate steps for both the insertion and removal of DGs and/or protecting groups (PGs), thus lengthening the synthetic pathways and producing chemical waste. Protocols that directly provide access to valuable products are therefore of particular importance. In this regard, methods that avoid the use of exogenous DGs offer improved atom‐economy compared to traditional procedures.

**Scheme 19:**
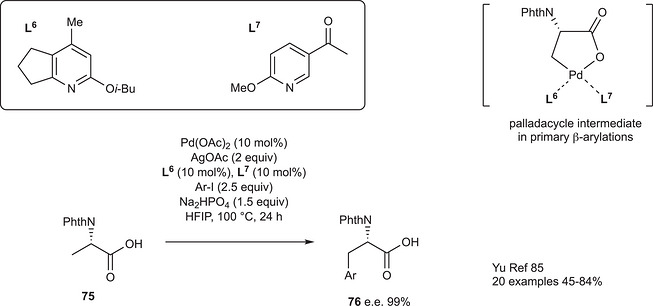
Pd(II)‐catalyzed **L^6^**/**L**7‐enabled β‐C(sp^3^)‐H arylation of Phth‐AlaOH.

In this context, Yu reported the successful carboxylate‐directed β‐arylation of Phth‐AlaOH by exploiting the simultaneous use of two pyridine‐type ligands (Scheme 19).^[^ ^85^ ^]^

Ligands (**L^6^**) and (**L^7^**) in hot HFIP represent the optimal combination for carrying out the β‐arylation reactions using the Pd(OAc)_2_/AgOAc catalyst system and the base Na_2_HPO_4_. The base is essential for generating the corresponding carboxylate, the true Pd‐coordinating group, while HFIP, as a solvent with excellent hydrogen‐bonding properties, is crucial to stabilize the catalytically active electrophilic Pd(II)‐species. Under these conditions, selective monoarylation of Phth‐AlaOH (**75**) to give (**76**) is the predominant outcome, as carboxylate‐directed bisligand activation favored primary β‐C(sp^3^)‐H arylation. Interestingly, the chirality of the α ‐center is preserved, but unfortunately, heteroaryl iodides proved to be incompatible under the optimized conditions.

**Scheme 20:**
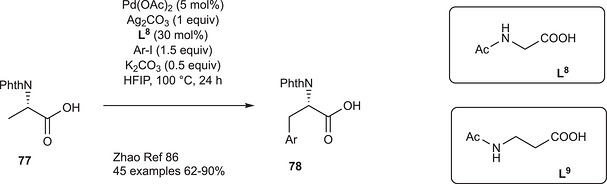
Pd(II)‐catalyzed **L^8^/L**9‐enabled β‐C(sp^3^)‐H arylation of Phth‐AlaOH.

Zhao's research group discovered that carboxylate‐directed Pd(II)‐catalyzed β‐C(sp^3^)‐H activation‐arylation of Phth‐Ala‐OH (**77**) could take advantage from the simple Ac‐GlyOH as a ligand. ^[^ ^86^ ^]^ Indeed, the mono‐protected amino acid ligand (**L^8^**) has allowed the preparation of many nonnatural phenylalanine derivatives (**78**) in moderate to excellent yields (Scheme 20). The ligand‐enabled direct β‐arylation protocol did not tolerate substrates with secondary β‐C(sp^3^)‐H as well as the Cbz protecting group.

The optimized conditions involved heating a solution of Phth‐AlaOH and Ar‐I (1.5 equiv) in HFIP at 110 °C for 24 h in the presence of a mixture of Pd(OAc)_2_ (5 mol%), Ag_2_CO_3_ (1 equiv), K_2_CO_3_ (0.5 equiv), and Ac‐GlyOH (30 mol%). At the same time van Gemmeren^[^ ^87^ ^]^ established that Ac‐β‐AlaOH (**L^9^**) had an effect comparable to Ac‐GlyOH (**L^8^**).

**Scheme 21:**
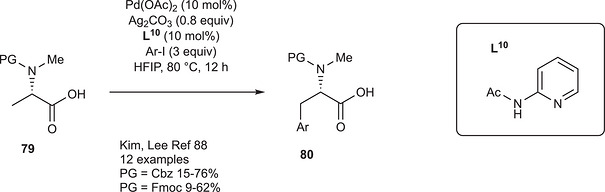
Pd(II)‐catalyzed **L^10^**‐enabled β‐C(sp^3^)‐H arylation of Cbz/Fmoc‐AlaOH.

More recently, aiming to obtain AAs derivatives equipped with PGs commonly employed in solid phase peptide synthesis (SPPS), ligand‐enabled β‐C(sp^3^)‐H arylation reactions on *N*‐Cbz and *N*‐Fmoc protected *N*‐metylalanine (**79**) have been investigated (Scheme 21).^[^ ^88^ ^]^ The monoprotected bidentate amino‐pyridine ligand (MPAP) (**L^10^**) was found to be crucial in accelerating the β‐C‐H activation step, leading to (**80**). Importantly, the method provided several Fmoc‐protected amino acid derivatives ready for SPPS, without any detectable racemization at the α ‐carbon. The optimal reaction conditions for β‐C(sp^3^)‐H arylation were: heating the substrate and ArI (3 equiv) in HFIP at 80 °C for 12 hours in the presence of (**L^10^**) (0.8 equiv), Pd(OAc)_2_ (10 mol%), Ag_2_CO_3_ (0.8 equiv). Diarylated products were not observed, while heteroaryl iodides and *N*‐Boc‐*N*‐methylalanine proved unsuitable.

## 4 Late‐Stage Functionalization of Peptides via β‐C‐H Arylation

LSF of natural products or medicinally active agents produces analogues that may have improved pharmacodynamics or pharmacokinetic properties. Many peptides' postsynthetic modifications are based on the transformation of “active” functional groups such as amino, hydroxyl, carboxyl and thiol. Moreover, aromatic C(sp^2^)‐H functionalization of Tyr, Trp, Phe, and His peptides has also been extensively investigated.

Inert β‐C(sp^3^)‐H bond activation‐arylation of specific α ‐amino acids in their complex environment have also been pursued. This section is focused on such a type of LSF, which inevitably poses challenges in terms of both chemo‐ and/or regioselectivity.

To organize all the reports collected on this topic, the criterion adopted is the same of Sections 2 and 3; therefore, sub‐section 4.1 is dedicated to methods exploiting amide‐linked DG, sub‐section 4.2 to methods taking advantage of the use of specific Pd(II) ligands, and sub‐section 4.3 collects reports performing postsynthetic modifications of “native” peptide motifs.

### 4.1 Site‐Selective β‐Arylations of Peptides Equipped with Bidentate DGs

Stereoselective Pd‐catalyzed, DG‐oriented secondary β‐C(sp^3^)‐H arylation of proline (Scheme 4) has also been applied to di‐and tri‐peptides containing AQ‐linked proline at the C‐terminus.

**Scheme 22:**
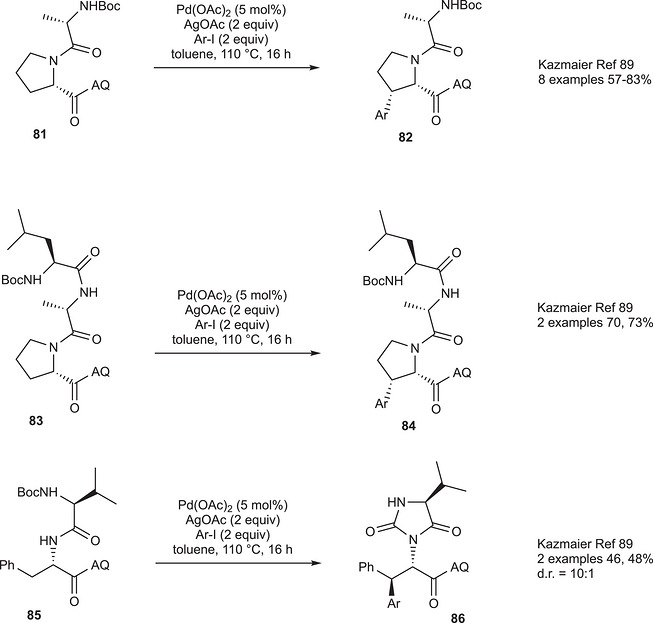
Pd(II)‐catalyzed, AQ‐directed secondary β‐C(sp^3^)‐H arylation of di‐ and tri‐peptides.

In 2016 Kazmaier^[^ ^89^ ^]^ reported the regio‐ and stereoselective β arylation of dipeptide Boc‐Ala‐Pro‐AQ (81) to give (82) and of tripeptide Boc‐Leu‐Ala‐Pro‐AQ (83) to give (84) (Scheme 22). The optimized conditions involved heating peptides and Ar‐I (2 equiv) in toluene at 110 °C for 16 hours in the presence of Pd(OAc)_2_ (5 mol%) and AgOAc (2 equiv). The method proved to be tolerant of secondary amides (peptide bonds) which, due to their propensity to bind Pd(II) forming chelate complexes, which are typically considered a major obstacle in palladium‐catalyzed C(sp^3^)−H functionalization of peptides. When the protocol was applied to Boc‐Val‐Phe‐AQ (85), the expected β‐arylated product was obtained as the corresponding imidazolidine 2,4‐dione derivative (86) due to cyclization reaction involving the Boc protecting group (Scheme 22).

**Scheme 23:**
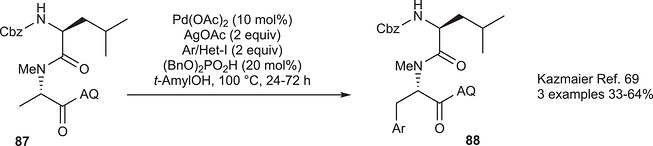
Pd(II)‐catalyzed, AQ‐directed primary β‐C(sp^3^)‐H arylation of dipeptide Cbz‐Leu‐(*N*‐Me)Ala‐AQ.

As anticipated in section 2.1. (Scheme 5) the dipeptide Cbz‐Leu‐(*N*‐Me)Ala‐AQ (87) was a suitable substrate for the C‐terminal (*N*‐Me)Ala β‐arylation giving (88) (Scheme 23).^[^ ^69^ ^]^ It is reasonable to assume that the *N*‐methyl favors the β‐arylation process, by suppressing palladium‐peptide chelate complexes.

**Scheme 24:**
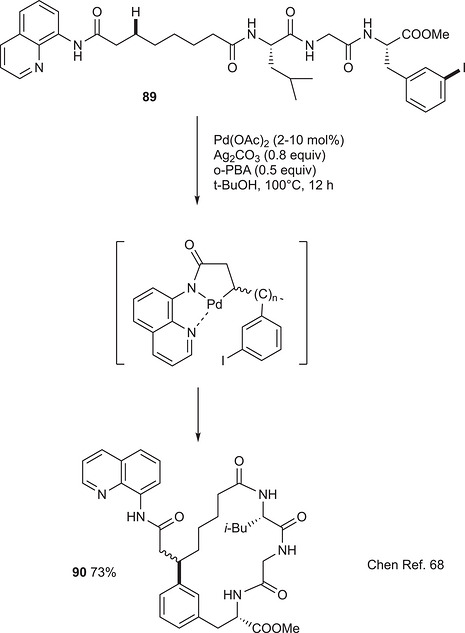
Pd‐catalyzed β‐C(sp^3^)‐H intramolecular arylation on AQ‐Sub‐Leu‐Gly‐(*m*‐I)PheOMe.

The palladium‐catalyzed, AQ‐directed β‐C(sp^3^)‐H arylation was also successfully accomplished intramolecularly to give cyclic peptides characterized by aromatic side chains incorporated into the macrocycle skeleton (cyclophane‐type structures) (Scheme 24).^[^ ^68^ ^]^

**Scheme 25:**
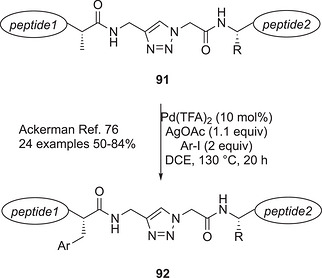
Pd‐catalyzed β‐C(sp^3^)‐H internal Tzl‐directed site‐selective β‐arylation.

As an example, the linear tripeptide AQ‐Sub‐Leu‐Gly‐(*m*‐I)PheOMe (**89**) featuring a *meta*‐iodinated Phe residue and an alkyl tail derived from AQ‐capped suberic acid at the *N*‐terminus, was smoothly cyclized to give 18‐membered *meta*‐cyclophane product (**90**) as a 1:1 diastereomeric mixture at the β carbon of the alkyl tail in excellent yield. The optimized conditions were: heating in *t*‐BuOH at 100 °C for 12 hours in the presence of Pd(OAc)_2_ (2–10 mol%), Ag_2_CO_3_ (0.8 equiv) and *ortho*‐phenyl benzoic acid (*o*‐PBA) (0.5 equiv). Computational analysis indicated that, compared to the intermolecular arylation pathway, the intramolecular arylation pathway is entropically favored. The highly efficient and operationally simple method demonstrated remarkable tolerance with respect to peptide composition, macrocycle size, cyclophane linker geometry, and choice of alkyl tail. In addition to suberic acid (Sub, C8), other alkyl diacids such as adipic acid (Adi, C6), pimelic acid (Pim, C7), and azelacid acid (Aze, C9) are also effective. Cyclization reactions generally proceeded cleanly, forming minimal side products, primarily small amounts of dimers, whose formation could be controlled by working under dilute conditions (5 mM). Notably, succinic acid (C4) and glutaric acid (C5) failed to give any product.

Peptide substrates incorporating modified nonaromatic amino acid units bearing prosthetic iodoaryl substituents on the side chains have also been selectively cyclized to form cyclophane‐braced peptide cycles of various sizes and shapes.

This strategy is not fully consistent with the review topic, since CHF does not involve the β‐C(sp^3^)‐H bond of an AA. However, as it represents a site‐selective β‐arylation of peptides equipped with the bidentate directing group (AQ), we have chosen to include it in this sub‐section.

Further example of macrocyclization of linear peptides via intramolecular b‐C(sp3)‐H arylation of amino acids is discussed in the sub‐section 4.3 (scheme 30).

Ackermann^[^ ^76^ ^]^ employed a 1,2,3‐triazole (Tzl) moiety as a peptide bond isostere and as a directing group for internal C─H arylation of (91) affording (92) with excellent chemo‐ and positional selectivity in a bioorthogonal manner (Scheme25). Extensive optimization studies identified DCE as the optimal solvent and Pd(TFA)_2_ as the catalyst of choice, enabling late‐stage diversification of internally functionalized peptides under racemization‐free conditions.

**Scheme 26:**
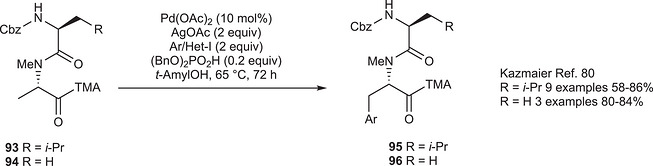
Pd(II)‐catalyzed, TMA‐directed primary β‐C(sp^3^)‐H arylation of Cbz‐Leu/Ala‐(*N*‐Me)Ala‐TMA.

As anticipated in section 2.5. (Scheme 14) both the dipeptide Cbz‐Leu‐(*N*‐Me)Ala‐TMA (93) and the Cbz‐Ala‐(*N*‐Me)Ala‐TMA (94) were suitable substrates for the C‐terminal (*N*‐Me)Ala β‐arylation giving peptides (95) and (96) respectively (Scheme 26).^[^ ^80^ ^]^

### 4.2 Ligand‐Controlled Site‐Selective β‐Arylations of Peptides

In 2021, Yao and colleagues^[^ ^90^ ^]^ reported a protocol for β‐C(sp^3^)‐H arylation of the C‐terminal Ala residue in peptides, using a strategy that exploits the cooperation between a bidentate DG and the substituted 2‐pyridinone ligand (L^11^).

**Scheme 27:**
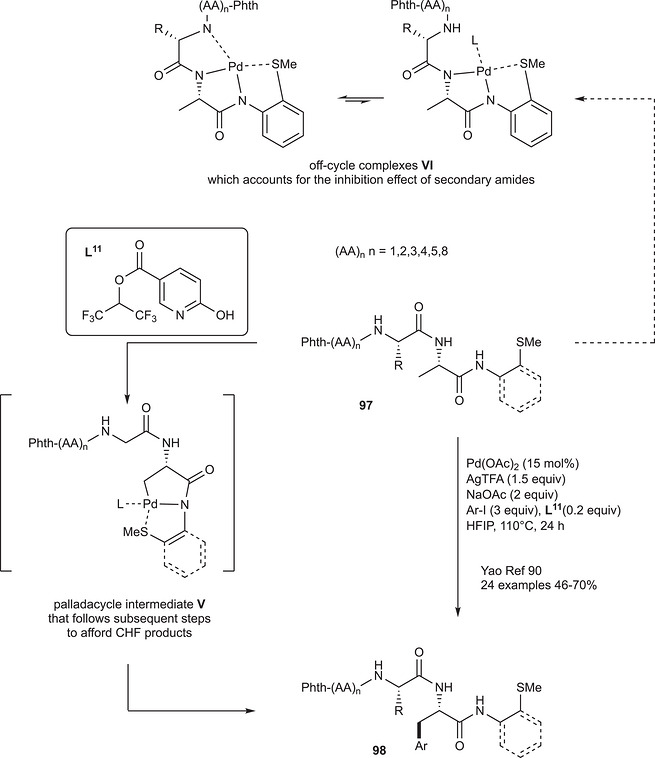
Pd(II)‐catalyzed **L^11^**‐enabled LSF on C‐terminal Ala of Phth‐AA_n_‐Ala‐MTEA.

Specifically, they identified 2‐(methylthio)ethylamine (MTEA) as the *N*,*S*‐bidentate DG which, in cooperation with (L^11^), lowers the activation energy of the C─H bond cleavage step (see Scheme 1). It was proposed that (L^11^), through steric effects, drives the Pd/peptide species toward the formation of the palladacycle intermediate (V), slowing the formation of the off‐cycle complexes (VI) (Scheme 27).

The optimal conditions involved heating the prefunctionalized peptides and Ar‐I (3.0 equiv) in HFIP at 110 °C for 24 hours in the presence of (L^11^) (0.2 equiv), Pd(OAc)_2_ (15 mol %), AgTFA (1.5 equiv), NaOAc (2 equiv).

Reactivity did not decrease with increasing peptide length, and 2‐thiomethylaniline (TMA) was also found to be a suitable bidentate ligand for directing β‐arylation. Under these conditions, a wide range of peptides (from tri‐ to dodecapeptides) (97) underwent site‐selective modification at the C‐terminal alanine, affording products (98) in good yields (Scheme 27). However, peptides containing residues with polar side chains or active C(sp^2^)‐H bonds proved to be incompatible with the conditions. Both the *N*‐linked protecting group (Phth) and the C‐linked auxiliary group (MTA) were removed sequentially under mild conditions: the latter via selective Boc‐activation of the most acidic *N*‐aryl amide, followed by amide cleavage with LiOH/H_2_O_2_.

**Scheme 28:**
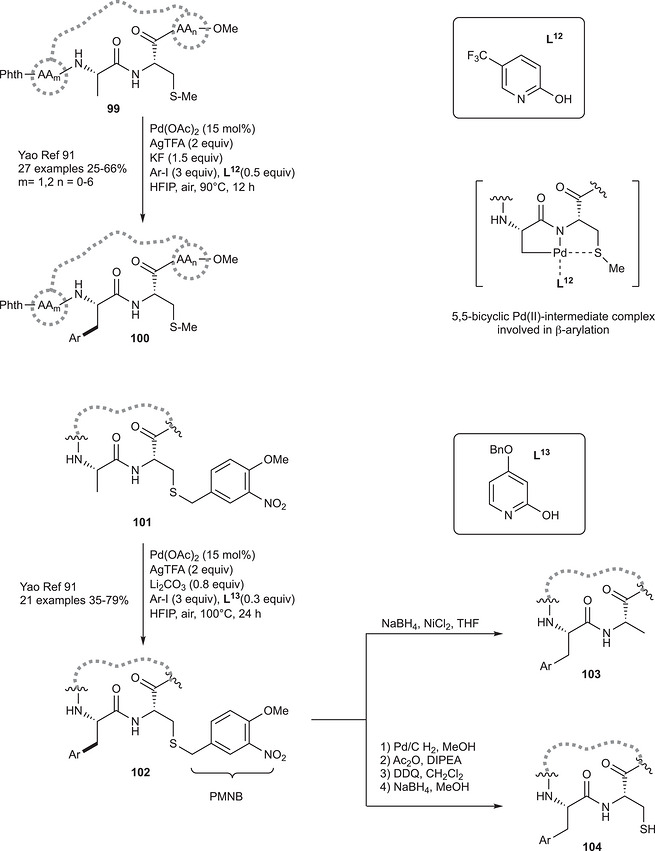
Cys‐directed, Pd(II)‐catalyzed, **L^12^**/**L**13‐enabled β‐C(sp^3^)‐H arylation of Ala at the internal position of peptides.

More recently, Yao and coworkers^[^ ^91^ ^]^ reported a strategy for direct internal peptide modification based on ligand‐enabled, *S*‐alkyl cysteine‐directed β‐C(sp^3^)‐H arylation. Specifically, using Cys^Me^ as the *N*,*S*‐bidentate directing group and 2‐pyridone (L^12^) as the Pd(II)‐ligand, a wide range of linear and cyclic peptides (99) were selectively β‐arylated on the Ala*_i_*_‐1_ side chain to successfully afford modified peptides (100) (Scheme 28). The proposed catalytic cycle proceeds via a palladacycle intermediate analogous to (V) in Scheme 27.

AA‐residues with nonpolar side chains were well tolerated, whereas other residues such as tyrosine (Tyr), threonine (Thr), and methionine (Met) showed moderate compatibility. A broad range of Ar‐I partners was effective, with the exception of *ortho*‐substituted derivatives, likely due to steric hindrance. Optimal conditions were heating peptide and Ar‐I (3 equiv) in HFIP at 90 °C for 12 h in the presence of ligand (L^12^) (0.5 equiv), Pd(OAc)_2_ (15 mol%), KF (1.5 equiv), AgTFA (2 equiv). Notably, replacing AgTFA with the more basic AgOAc as iodide scavenger lowered yields due to the transformation of Cys^Me^ to dehydroalanine (Dha) via β‐elimination.

The search for removable Cys^Me^ surrogates led to the identification of 4‐methoxy‐3‐nitrobenzyl (PMNB) as a protecting group that is stable under β‐arylation conditions but offers the opportunity for further transformations. By slightly modifying the previous reaction conditions and employing the (L^13^) ligand, cyclic peptides bearing the Ala‐Cys^PMNB^ motif (101) were converted into derivatives (102) in modest to high yields. PMNB removal could be achieved in two ways: i) desulfurization with nickel boride to give (103), or ii) recovery of the unprotected Cys residue (104) via multistep sequence involving Pd/C‐catalyzed hydrogenation of NO_2_, acetylation of NH_2_ by Ac_2_O, benzylic oxidation by DDQ, and S─S bond cleavage by NaBH_4_.

### 4.3 Site‐Selective β‐Arylations of “Native” Peptide Motifs

**Scheme 29:**
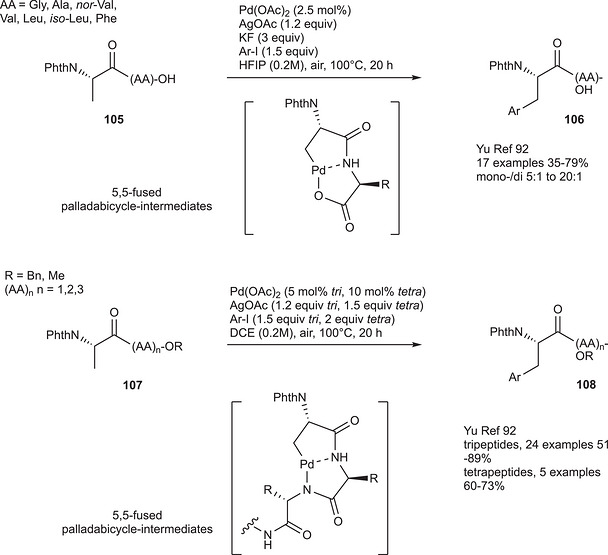
Late‐stage backbone‐assisted β‐C(sp^3^)‐H arylation on the *N*‐terminus Phth‐Ala.

In 2014 Yu and coworkers^[^ ^92^ ^]^ demonstrated that native amino acids incorporated into the peptide backbone could bind to Pd(II) via *N*,*O*‐ or *N*,*N*‐bisdentate coordination and promote activation‐arylation of nearby β‐C(sp^3^)‐H bonds on the adjacent amino acid Phth‐Ala*_i_*_‐1_ (Scheme 29).

It has been proposed that backbone‐facilitated C─H functionalization (CHF) on Phth‐Ala in dipeptides proceeds through 5,5‐fused palladabicycle‐intermediates involving the *N* atom of the adjacent peptide bond as an L‐type ligand and the *O* atom of the carboxylate as an X‐type ligand. Under the optimized conditions [(Ar‐I (1.5 equiv), Pd(OAc)_2_ (2.5 mol%), AgOAc (1.2 equiv), KF (3 equiv), HFIP 100 °C, 20 hours], dipeptides of general formula Phth‐Ala‐AA‐OH (105) were arylated at the *N*‐terminus with a wide range of aryl iodides to give (106) in good yields.

Furthermore, selective β‐arylation on Phth‐Ala in tri‐ and tetra‐peptides of general formula (107) gave the desired products (108) in good yields, performing the reaction in 1,2‐dichloroethane (DCE) and making small modifications to the reaction conditions. In these cases, it has been proposed that CHF proceeds through 5,5‐bicylic‐Pd(II)‐intermediate complexes involving both *N* atoms of consecutive peptide bonds, the first as an L‐type ligand and the second as an X‐type ligand.

In dipeptides, the C‐terminus provides the carboxylate group as the native directional group, whereas in longer peptides this role is played by the second amide bond in the peptide sequence (Scheme 29).

Importantly, sequential C─H functionalizations of peptides of different lengths have been shown to enable multiple structural modifications at sites beyond the *N*‐terminus.

**Scheme 30:**
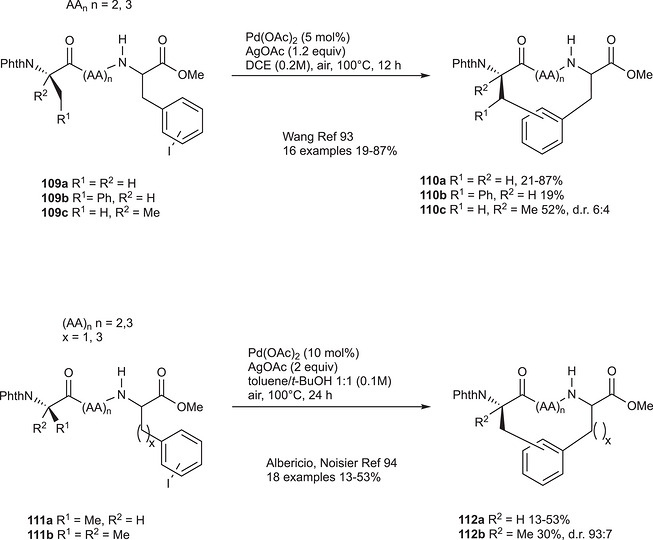
Late‐stage backbone‐assisted β‐C(sp^3^)‐H intramolecular arylation.

In 2017, Wang and coworkers^[^ ^93^ ^]^ and, independently, Albericio and coworkers^[^ ^94^ ^]^ hypothesized that the intermolecular arylation strategy disclosed by Yu^[^ ^92^ ^]^ could also operate intramolecularly, thereby enabling the formation of cyclic peptides, compounds of considerable importance as peptide‐based drugs and biological probes (Scheme 30). It was predicted that the usual 5,5‐fused palladabicycle‐intermediates could react intramolecularly with an iodoaryl side‐chain of AA residues introduced in the same peptide sequence to provide a structurally unique building block. Indeed, a variety of cyclic peptides of general formula (**110**) containing unique β‐C‐Ar crosslinks between the β‐carbon of aliphatic amino acids and the aromatic ring of Phe/Trp were synthesized through a Pd‐catalyzed C(sp^3^)‐H activation‐arylation process in moderate to good yields. Under Wang's optimized conditions [Pd(OAc)_2_ (5 mol%), AgOAc (1.2 equiv), in DCE at 100 °C] several tetrapeptides and selected pentapeptides of general formula (**109**) were successfully converted into cyclic peptides with *i,i *+ 3 and *i,i *+ 4 topologies, respectively, in moderate to good isolated yields (Scheme 30).

Peptides with *N*‐terminal residues other than Ala proved less suitable; in particular, α‐aminoisobutyric acid (Aib) provided the predicted cyclic peptide (110c) as a 6:4 diasteromeric mixture, while the cyclization yield with Phe (110b) was only 19%.

Furthermore, tetra‐ and penta‐peptides with *para*‐ or *ortho*‐I‐Phe were poor substrates; in these cases, however, the insertion of a proline (Pro) residue, a known conformational turn‐inducing element, was found to have beneficial effects on cyclization efficiency.

Similarly, by only slightly modifying reaction conditions [Pd(OAc)_2_ (10 mol%), AgOAc (2 equiv), peptides in 0.1 M *t*‐BuOH/toluene 1:1 at 100 °C for 24 hours], Albericio and Noisier^[^ ^94^ ^]^ succeeded in the cyclization of linear tetra‐ or pentapeptides (111) to give (112). Note that, unlike the result found by Wang^[^ ^93^ ^]^ for the linear tetrapeptide Phth‐Aib‐Gly‐Gly‐(*m*‐I‐Phe)‐OMe (general formulae 109c to 110c 6:4 d.r), the internal CHF of tetrapeptide Phth‐Aib‐Ser(*t*Bu)‐Gly‐(*m*‐I‐Phe)‐OMe (general formulae 111b to 112b 93:7 d.r) occurred with excellent diastereoselectivity under Albericio conditions.

It is important to note that both research groups were able to transpose their methods in solution onto the solid phase. With a wide range of protected side‐chain functional groups being well tolerated, the methods pave the way for easily accessing cyclic peptide libraries.

**Scheme 31:**
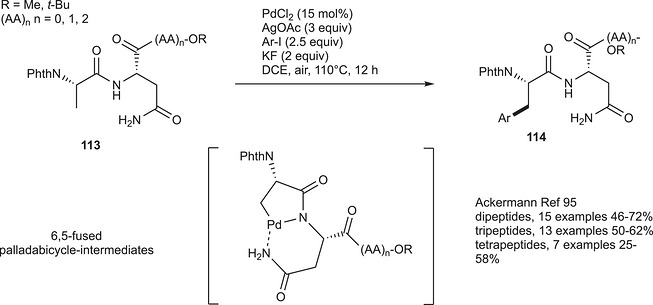
Asn‐directed Late‐stage β‐C(sp^3^)‐H arylation on the *N*‐terminus Phth‐Ala.

In 2020, a report by Ackermann *et al.* ^[^ ^95^ ^]^ demonstrated that the unmodified side chain of asparagine (Asn) acted as an internal bidentate DG to produce a Pd(II)‐coordinated complex that was effective in late‐stage peptide β‐C(sp^3^)‐H arylation (Scheme 31). Under optimized conditions [PdCl_2_ (15 mol%), AgOAc (2.5 equiv), Ar‐I (2.5 equiv), KF (2 equiv), DCE 110 °C, 12 hours], tri‐ and tetra‐peptides of general formula (113) underwent selective monoarylation of β‐C(sp^3^)‐H bonds at the *N*‐terminus to give (114). A 6,5‐fused palladabicycle‐intermediate has been proposed to arise from Asn‐directed C─H activation via a CMD mechanism.

The strategy, which enables selective arylation on Phth‐Ala, shows broad tolerance toward Ar─I bearing either electron‐donating/electron‐withdrawing groups, as well as biphenyl or naphthyl iodides.

**Scheme 32:**
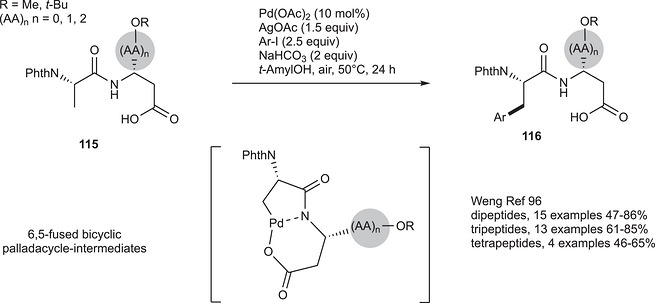
Asp‐directed Late‐stage β‐C(sp^3^)‐H arylation on the *N*‐terminus Phth‐Ala.

The following year, an important contribution to the topic came from the Weng's research group.^[^ ^96^ ^]^ They reported that the side chain and backbone of Asp can serve as an endogenous DG in late‐stage β‐C(sp^3^)‐H arylation (and alkynylation) of peptide sequences (Scheme 32). Indeed, the aspartic amino acid residue acts as an *N*,*O*‐bidentate ligand, leading to the formation of a fused 6,5‐palladacycle intermediate analogous to that proposed for the *N*,*N*‐bidentate ligand Asn.^[^ ^95^ ^]^ In this case, however, Pd(OAc)_2_ was used as a catalyst under milder reaction conditions (*t*‐AmylOH at 50 °C).

The reactions proceeded efficiently across di‐, tri‐, and tetra‐peptide substrates (115) providing the corresponding modified peptides (116) in moderate to good yields.

**Scheme 33:**
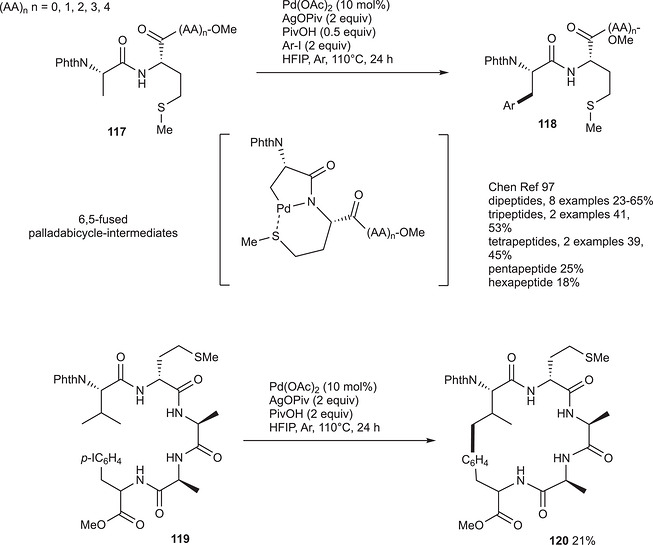
Met‐directed Late‐stage β‐C(sp^3^)‐H arylation on the *N*‐terminus Phth‐Ala.

In 2022, Chen and coworkers^[^ ^97^ ^]^ demonstrated that the unmodified side chain and backbone of methionine (Met) behaved as an endogenous *N*,*S*‐bidentate DG (Scheme 33). Plausibly, a flexible six member Pd(II)‐coordinated complex is first formed, promoting β‐C(sp^3^)‐H arylation of the *N*‐terminal Ala residue in short peptides. The reaction conditions involved heating in HFIP at 110 °C a mixture of the peptide substrate of general formula (117), Ar‐I (2 equiv), Pd(OAc)_2_ (10 mol%), AgOPiv (2 equiv), PivOH (0.5 equiv). The desired monoarylated products (118) were obtained along with minor amounts of β,β‐diarylation by‐products. Electron‐rich aryl iodides gave higher arylation yields than electron‐deficient ones, although overall yields decreased progressively with increasing peptide length. Furthermore, replacing the Phth‐Ala‐Met motif with Phth‐Val‐Met redirected the arylation to the γ‐C(sp^3^)‐H bond of Val. This finding was elegantly exploited in the Met‐directed intramolecular γ‐methyl C─H arylation of (119), which delivered the cyclic pentapeptide (120).

Meanwhile, Yao and coworkers^[^ ^98^ ^]^ confirmed that the Phth‐Ala‐Met motif was suitable for CHF via a possible six member *N,S*‐palladium ring, but they observed a drastic decrease in reactivity when the Ala residue was shifted from the *N*‐terminus to an internal position. This loss of activity was attributed to the inhibitory effect of secondary amides.

**Scheme 34:**
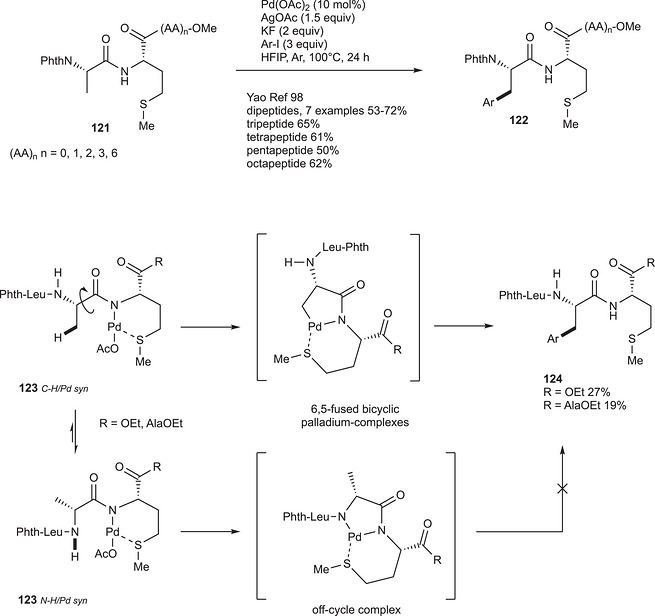
Met‐directed Late‐stage β‐C(sp^3^)‐H arylation on the *N*‐terminus Phth‐Ala vs internal Ala.

Under optimized conditions involving heating to 100 °C in HFIP of a mixture of linear peptide (**121**) and Ar‐I (3 equiv) in the presence of the catalyst system, the corresponding arylated derivatives (**122**) were obtained with excellent position selectivity and satisfactory yields. (Scheme 34). Instead, substrates (**123**) gave the predicted arylated compounds (**124**) only in poor yields. The authors hypothesized the involvement of two 6,5‐fused palladium bicyclic complexes: one resulting from the (**123**) conformer with a *syn* C─H/Pd relationship, and a competitive off‐cycle complex derived from the conformer with a *syn* N─H/Pd relationship (Scheme 34).

**Scheme 35:**
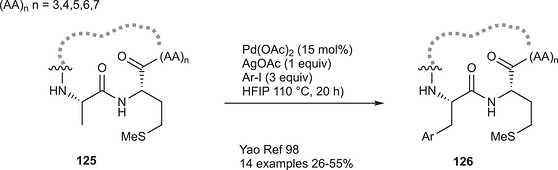
Met‐directed Late‐stage β‐C(sp^3^)‐H arylation on head‐to‐tail macrocyclic peptides.

Given the restricted conformational flexibility of cyclic peptides compared to the linear counterparts, the authors were confident that the inhibitory effect of the peptide backbone could be mitigated by embedding the Ala‐Met sequence into the cyclic peptides (**125**) (Scheme 35). Indeed, under the standard conditions, a wide range of head‐to‐tail cyclic peptides (penta‐ to nonapeptides) (**125**) containing the L‐Ala‐L‐Met sequence underwent β‐C(sp^3^)‐H arylation of Ala to give (**126**) in moderate to good yields with excellent position selectivity. Intriguingly, in a cyclic hexapeptide where L‐Met served as directing group, D‐Ala failed to undergo arylation.

## 5 Summary and Outlook

In this mini review, we have traced the evolution over the past decades of strategies for synthesizing unnatural amino acids or peptides via multiple palladium‐catalyzed processes. Our interest has been restricted to methodologies that generate nonnatural derivatives via new C─C sigma bond formation between β‐C(sp^3^) of AAs and C(sp^2^) of Ar─I. A unifying feature of all the discussed strategies is the use of C─H functionalization (CHF) as the key step.

Starting from methods using AAs covalently linked to bidentate directional groups (DGs), we moved to ligand‐enabled reaction protocols and then to peptides LSF.

LSFs include both methods exploiting exogenous DGs/Ls and protocols working on “native” peptides, which can be further divided into skeleton‐assisted CHF and side chain‐assisted CHF.

Given the remarkable importance of cyclic peptides as privileged scaffolds in peptide‐based drug research, we also highlighted the few strategies developed so far that provide them via β‐C(sp^3^)‐H internal arylation reactions.

Despite the considerable progress in recent years, several challenges remain. For CHF of single amino acids: i) most arylation methods are effective on primary or secondary β‐C(sp^3^)‐H bonds, while the CHF of valine occurs at the γ‐position preventing the obtaining of AA‐derivatives with the quaternized β‐carbon; ii) desymmetrization of the prochiral carbon of 2‐aminoisobutyric acid (Aib) remains an unsolved problem; iii) the requirement for prefunctionalized AAs or effective ligands can complicate protocols, due to the harsh conditions usually required to isolate arylated AAs and the extra work required to recover structurally elaborated ligands, limiting atom and step economy.

As regards CHF of peptides: i) current methods predominantly target the *C*‐ and *N*‐termini, whereas CHF on internal amino acid residues remains challenging due to the need for suitable side chains to act as internal DG; ii) many protocols often require prolonged heating at high temperatures in expensive or environmentally unfriendly solvents; iii) skeleton‐assisted, site‐selective CHF often suffers from inhibition by secondary amides (peptide bonds).

Advances in these areas are highly desirable, as they could unlock new opportunities for the simplified synthesis of complex, unnatural peptides for applications in peptide‐based therapeutics.

## Conflict of Interest

The authors declare no conflicts of interest.
